# Assessment of postoperative pain management and comparison of effectiveness of pain relief treatment involving paravertebral block and thoracic epidural analgesia in patients undergoing posterolateral thoracotomy

**DOI:** 10.1186/s13019-019-0901-3

**Published:** 2019-04-16

**Authors:** Małgorzata Edyta Wojtyś, Józef Wąsikowski, Norbert Wójcik, Janusz Wójcik, Piotr Wasilewski, Piotr Lisowski, Tomasz Grodzki

**Affiliations:** 1 Department of Thoracic Surgery and Transplantation, Pomeranian Medical University, Szczecin, Poland; 2 Students’ Scientific Circle of the Department of Thoracic Surgery and Transplantation, Pomeranian Medical University, Szczecin, Poland; 3 The Department of Anesthesiology and Intensive Therapy, Prof. A. Sokołowski Specialist Hospital, Szczecin, Poland

**Keywords:** Regional analgesia, Posterolateral thoracotomy, Assessment of pain management quality, Epidural analgesia, Paravertebral block

## Abstract

**Background:**

TEA (thoracic epidural analgesia) is considered a basic method of analgesia used in thoracic surgeries. PVB (paravertebral block) is an alternative method. The thesis compares effectiveness of both methods in postoperative analgesia with particular focus on assessment of the postoperative pain management quality.

**Methods:**

The study involved 2 groups of patients, each consisting of 30 patients undergoing posterolateral thoracotomy. The study group involved patients anesthetized applying PVB method, while the control group involved patients anesthetized with TEA. Hemodynamic and respiratory parameters as well as severity of pain assessed using NRS (numeric rating scale) during the first 3 days after the surgery, number of days of hospitalization, and the need to use additional pain relievers were taken into account in both groups. Evaluation of postoperative pain management quality was performed applying Clinical Quality Indicators in Postoperative Pain Management.

**Results:**

No statistical significance was demonstrated between the groups in respect of hemodynamic and respiratory parameters values, the need to use additional pain relievers and the number of days of hospitalization. There was no statistically significant difference between the groups in respect of general assessment of pain management quality, except for the assessment of the lowest level of pain within the last 24 h of measurement. This result in TEA group was statistically significantly lower than the one in PVB group (*p* = 0.019).

**Conclusions:**

In the assessment of postoperative pain management quality both analyzed methods are statistically significantly different only in the category of “lowest level of pain within the last 24 hours of measurement”, to the benefit of TEA group. No statistically significant difference has been observed between the two study groups with respect to the remaining parameters.

**Trial registration:**

KB-0012/71/15. Date of registration 22 June 2015.

## Background

Thoracotomy is a type of a surgery that involves one of the most perceptible pains during the postoperative period [1]. Pain after the chest surgery usually lasts approximately 72-96 hours and is more severe than pain after the surgeries of other body areas. This is related, among others, to the fact that the chest is moving as a whole, so it also involves the operated area [2]. Pain after the thoracic surgeries decreases diaphragmatic and other respiratory muscles function, leading to respiratory disorders, including hypoxia, pulmonary infections and cardiologic complications [1, 3].

Due to the fact that during posterolateral thoracotomy numerous layers of the chest wall are being cut and dissected, and the dissection takes place within the lung itself and its components (bronchial tubes, vessels, nerves), there are many sources and mechanisms of the post-thoracotomy pain. This not only results from nociceptive mechanisms, but also involves neuropathic component resulting from minor or larger damage to intercostal nerves or other nerves during the surgery. Pulling of visceral or vagus nerves as well as the presence of drain in the pleural cavity after the operative procedure also cause the sensation of pain [4]. Methods involving regional analgesia are often used in treatment of pain post thoracotomy and post thoracoscopic surgeries.

Thoracic epidural analgesia (TEA) is commonly used. The use of the thoracic epidural analgesia in the case of thoracic surgical procedures reduces the stress response to a surgical injury; it favorably affects systemic homeostasis in respect of hormonal response, functions of the immune, coagulation and alimentary system. Epidural analgesia may be accompanied by adverse reactions such as hypotonia, urine retention, respiratory muscles weakness. Other possible complications include epidural abscess, hematoma in the epidural area. In extreme cases, it may result in paraplegia due to damage to the spinal cord, what is observed extremely rarely (below 0.02%) [1, 3, 5]. TEA or other methods of regional analgesia are also used, among others, in the case of operations involving the thoracic glands, post vertebral column stabilization procedures or urologic operations [6–8].

There are quite numerous contraindications to TEA, such as clotting disturbances caused by intake of anticoagulant medications (e.g. Low Molecular Weight Heparin, Clopidogrel). If a patient is treated with Low Molecular Weight Heparin, epidural block may be performed after 12 hours of heparin administration. According to some authors, TEA is ineffective in more than 12% of patients, in particular in the case of difficult anatomic conditions [3].

Paravertebral block (PVB) within the chest area is considered an alternative method to TEA, among others, in the case of contraindications to TEA. PVB enables analgesia of the chest only on the side being operated, and the analgesia involves only the area of surgical wound and additionally few segments above and few below the wound, depending on the volume of the regional analgesic agent injected [9, 10].

To reach higher precision, ultrasonography control or nerves stimulation are more often used while applying the paravertebral block [3, 11]. The block can also be applied intraoperatively under direct vision. PVB is contraindicated in the case of difficult anatomic conditions, or infection in the area of planned block application [3]. Blood clotting disturbances do not constitute a contraindication to paravertebral block [8].

Other methods of regional analgesia, such as intercostal block or intrapleural administration of pain relievers are also applied [4].

Although PVB was belived as effective on pain relief as TEA, there are limited evidence on postoperative analgesia for posterolateral thoracotomy. In this study, we goaled to compare the effectiveness between TEA and PVB by measuring numeric pain rating scale (NRS) and clinical quality index (CQI) during the first 3 postoperative days.

Primary purposes of the study involved:

1. Comparison of the effectiveness of PVB and TEA in treatment of postoperative pain in patients undergoing posterolateral thoracotomy.
2. Assessment of postoperative pain management quality based on Clinical Quality Indicators in Postoperative Pain Management in patients who underwent posterolateral thoracotomy and were treated with PVB or TEA.

## Methods

Informed consent to participate in the study was obtained from each patient. Patients were provided with detailed description of benefits, side effects and complications related to both types of analgesia. Ethics Committee of the Pomeranian Medical University in Szczecin approved the study conduct (KB-0012/71/15).

All surgeries were performed applying 10-12 cm long thoracotomy without the use of VATS at one Department of Thoracic Surgery, as consecutive cases. Operations in both groups had similar scope - from tumor resection through segmentectomy, lobectomy through pneumonectomy.

The study involved two groups of patients, each consisting of 30 patients of both sexes, above 18 years of age. Patients were randomized to particular groups (simple randomization). Patients from both groups had no contraindications to application of any of the postoperative analgesia methods.

Group 1 involves patients who had PVB, while group 2 involves patients who were treated with TEA

### Paravertebral block (PVB)

PVB was performed in the case of patients premedicated with 7.5 mg oral midazolam. It was performed in sitting position, according to slightly modified technique provided for by Cousin and Bridenbaugh [12]. Epidural catheter was placed in the paravertebral area at the level of the lower edge of the spinous process Th4/Th5 ca. 2.5 cm laterally from the spinous process, depending on the planned surgical incision. Catheter was placed in the paravertebral area at a depth of 3-4 cm. Thereafter, 0.5% bupivacaini in the total volume from 15 to 25 ml was injected prior to analgesia induction. After completion of the procedure, patients were given additional 15 ml of 0.25% bupivacaini. After being transferred to the department of thoracic surgeries the patients were given continuous infusion of 0.25% bupivacaini at the dose of 0.1 ml per kilogram of body weight (5-8 ml per hour).

### Thoracic epidural analgesia (TEA)

The second group involved patients who, prior to the posterolateral thoracotomy, were given epidural analgesia performed in the sitting position applying the “hanging drop” technique at the level of Th5/Th6 in the medial body line. Upon identification of the epidural area, an epidural catheter was inserted through Tuohy needle about 4 to 5 cm into the epidural space. Thereafter, test dose of 2 ml of 1% lidocaine was injected to exclude spinal anesthesia. Then from 6 to 8 ml of 0.5% bupivacaini with 0.05 mg of fentanyl was injected. Analgesia induction (in the case of both groups of patients) involved intravenous injection of propofol at the dose of 2 mg per kilogram of the body weight, fentanyl 0.1 mg, cisatracurium at the dose of 0.15 mg per kilogram of the body weight. Patients were intubated with two-channel tube. Analgesia was maintained with respiratory mixture of sevoflurane, nitrous oxide and oxygen in commonly used concentrations. Upon completion of the procedure, patients were additionally given 6-8 ml of 0.5 % bupivacaini to epidural catheter. After being transferred to the Department of Thoracic Surgery the patients were given continuous infusion of 0.125% bupivacaini in the volume of 0.1 ml per kilogram of the body weight (6-8 ml per hour) and fentanyl at the dose of 2 micrograms per ml for 72 hours.

All patients were administered nasal oxygen with the flow of 3 liters per minute, for 24 hours. Patients were provided with continuous monitoring of vital signs: saturation (SpO2), systolic and diastolic blood pressure, pulse, number of breaths, consciousness.

Patients from each of the groups underwent posterolateral thoracotomy and resection of pulmonary parenchyma mainly due to lung tumor, but also due to the presence of emphysematous bulla, diagnosed based on the typical diagnostic methods, the same for both groups.

Patients qualified to the study were randomized to particular groups (simple randomization).

Exclusion criteria included: failure to obtain the patient’s consent, clotting disturbances making it impossible to perform regional analgesia, infection in the area of planned catheter insertion, significant spinal column deformation, difficulties with proper pain severity assessment and T4-infiltrating tumors with continuous pain.

The study was performed in each of the groups according to the same schedule. Measurements had been performed for first three days after the procedure.

Each group had evaluations of SpO2 every 4 hours, arterial blood pressure and pulse every 4 hours, assessment of pain (NRS) every 4 hours, anesthetized area every 4 hours, the use of additional pain relievers, Clinical Quality Indicators in Postoperative Pain Management.

Values regarding current pain in NRS scale (0-10 pints), the highest level of pain within the last 24 hours (0-10 pints), the lowest level of pain within the last 24 hours (0-10 pints) were marked on the linear scale in the same intervals in the scale of 0-10 points. Satisfaction with the manner of postoperative pain management was assessed using the scale of 1-10 points.

Clinical Quality Indicators in Postoperative Pain Management scale was used to assess satisfaction with postoperative pain relieving treatment and quality of the postoperative pain management.

Clinical Quality Indicators in Postoperative Pain Management scale allows to assess satisfaction with postoperative pain relieving treatment and quality of the postoperative pain management, however Visual Analog Scale (VAS) and Numeric Pain Rating Scale (NRS) are the simplest and most often used to assess pain. Clinical Quality Indicators in Postoperative Pain Management, a scale developed by Swedish author, Eva Idvall, enables assessment of many various aspects of postoperative pain management quality [13]. This scale was most of all developed to assess satisfaction with nursing care, but is also used to perform general assessment of postoperative pain management quality. It was translated, among others, to Polish and adopted to local conditions. Assessment with the use of Clinical Quality Indicators in Postoperative Pain Management scale is performed according to 5-point Likert scale, where 1 point means “strongly disagree”, and 5 points mean “strongly agree”. This scale involves also assessment of the highest and the lowest level of pain during the postoperative period within the last 24 hours, assessment of current postoperative pain, similarly to NRS (in the scale of 0-10), assessment of general satisfaction with postoperative pain management in the scale of 1-10 [14].

Anesthetized area, i.e. extent of the sensory blockade, was determined based on the number of anesthetized spinal segments according to the anatomic scheme.

Collected data was subject to statistical analysis performed with the use of SPSS package (SPSS Inc., Chicago IL, USA).

T-test for dependent groups and Mann-Whitney U test were used in statistical analysis. Statistical calculations were performed applying the statistical significance of 0.05.

## Results (Table 1)

The studied groups have not demonstrated statistically significant difference in terms of age and sex - *p* = 0.74 and *p* = 0.58, respectively.

**Table 1:** Demographic data of the study groups

Group	PVB - group 1 - number (%)	TEA- group 2 -number (%)	Total number (%)
Age in years number of persons (%)			
20–50	3 (10)	5 (16.7)	8 (13.3)
51–70	21 (70)	19 (63.3)	40 (66.7)
Above 70	6 (20)	6 (20)	12 (20)
Sex M/F	21/9 (70/30)	19/11 (63.3/36.7)	40/20 (66.7/33.3)

In the first group indication for surgery constituted: in the case of 14 patients – primary lung cancer, in the case of 4 patients – metastatic tumors, in the case of 3 patients – pleural mesothelioma, while in the case of 9 persons non-neoplastic diseases, such as recurrent spontaneous pneumothorax, benign tumors, pulmonary tuberculosis. In the second group primary lung cancer constituted indication for thoracotomy in the case of 11 persons, metastatic tumors in 4 patients, mediastinal tumors in 2 patients, while non-neoplastic diseases in 13 persons.

In the first group 11 lobectomies, 2 bilobectomies, 1 pneumonectomy, 2 segmentectomies, 7 tumor resections and 6 other surgeries (e.g. explorative thoracotomy, decortication, pleurectomy) were performed.

In the second group 7 lobectomies, 6 segmentectomies, 11 tumor resections and 6 other surgeries, such as decortication, explorative thoracotomy were performed. Range of mesothelioma operations were similar to the decortications or pleurectomy for non-neoplastic diseases.

Results of hemodynamic parameters measurements in the study group presents Table 2.

**Table 2:** Hemodynamic parameters: blood pressure (systolic (s) and diastolic (d)), pulse, arterial oxygen saturation as well as number of days of hospitalization in particular (PVB, TEA) groups during 3 days after surgery

	Group											Mann-Whitney U test	P
	PVB			TEA			Total						
	Mean	N	Standard deviation	Mean	N	Standard deviation	Mean	N	Standard deviation	Minimum	Maximum		
a blood pressure-s- (every 4 h) 1st day	122.10	30	11.25	119.13	30	13.50	120.62	60	12.41	98.00	148.00	360.50	.186
b blood pressure-d- (every 4 h) 1st day	69.30	30	8.89	66.83	30	7.64	68.07	60	8.31	53.00	87.00	369.50	.233
a blood pressure-s- (every 4 h) 2nd day	122.37	30	13.31	120.00	30	16.43	121.18	60	14.87	88.00	156.00	386.00	.344
b blood pressure-d- (every 4 h) 2nd day	70.07	30	10.79	67.47	30	7.64	68.77	60	9.36	48.00	87.00	386.00	.344
a blood pressure-s- (every 4 h) 3rd day	129.17	24	15.96	123.91	23	12.92	126.60	47	14.64	101.00	160.00	242.00	.469
b blood pressure-d- (every 4 h) 3rd day	72.50	24	10.16	72.09	23	8.81	72.30	47	9.42	55.00	89.00	273.50	.957
pulse 1st day	81.80	30	10.53	79.93	30	10.76	80.87	60	10.59	56.00	104.00	408.00	.534
pulse 2nd day	84.07	30	8.63	84.56	30	10.07	84.31	60	9.30	64.00	105.00	443.50	.923
pulse 3rd day	84.04	24	9.93	85.09	23	9.83	84.55	47	9.79	66.00	108.00	262.00	.765
SaO2 1st day	97.62	29	1.99	97.01	30	1.62	97.31	59	1.82	92.00	100.00	328.50	.102
SaO2 2nd day	95.18	17	2.30	95.20	20	2.50	95.19	37	2.38	90.00	100.00	160.00	.758
SaO2 3nd day	95.71	7	0.95	95.13	8	2.85	95.40	15	2.13	90.00	99.00	23.50	.595
number of days of hospitalization after surgery	7.47	30	3.70	6.30	30	1.95	6.88	60	2.99	3.00	23.00	354.00	.150

Tables 3, 4 and 5 present differences in values of arterial blood pressure, pulse and saturation on particular days of measurements in PVB and TEA groups, and in both groups altogether.

**Table 3:** Results of measurements of arterial blood pressure (systolic (s) and diastolic (d)), pulse, and saturation in both groups on particular days

Statistics for dependent groups					t-test	Degrees of freedom	p
Comparison of hemodynamic parameters between particular days after surgery in all patients (both groups altogether)	Mean	N	Standard deviation	Standard error of the mean			
a blood pressure-s- (every 4 h) 1st day	120.62	60	12.41	1.60	−0.31	59	.761
a blood pressure-s- (every 4 h) 2nd day	121.18	60	14.87	1.92			
a blood pressure-s- (every 4 h) 1st day	121.15	47	12.81	1.87	−2.32	46	.025
a blood pressure-s- (every 4 h) 3rd day	126.60	47	14.64	2.14			
a blood pressure-s- (every 4 h) 2nd day	120.98	47	15.66	2.28	−2.32	46	.025
a blood pressure-s- (every 4 h) 3rd day	126.60	47	14.64	2.14			
b blood pressure-d- (every 4 h) 1st day	68.07	60	8.31	1.07	−0.67	59	.504
b blood pressure-d- (every 4 h) 2nd day	68.77	60	9.36	1.21			
b blood pressure-d- (every 4 h) 1st day	67.81	47	8.33	1.22	−3.16	46	.003
b blood pressure-d- (every 4 h) 3rd day	72.30	47	9.42	1.37			
b blood pressure-d- (every 4 h) 2nd day	68.00	47	9.85	1.44	−3.04	46	.004
b blood pressure-d- (every 4 h) 3rd day	72.30	47	9.42	1.37			
pulse 1st day	80.87	60	10.59	1.37	−3.39	59	.001
pulse 2nd day	84.31	60	9.30	1.20			
pulse 1st day	80.57	47	10.26	1.50	−2.76	46	.008
pulse 3rd day	84.55	47	9.79	1.43			
pulse 2nd day	83.83	47	9.16	1.34	−0.73	46	.468
pulse 3rd day	84.55	47	9.79	1.43			
SaO2 1st day	97.19	37	1.69	0.28	5.40	36	.000
SaO2 2nd day	95.19	37	2.38	0.39			
SaO2 1st day	97.40	15	2.03	0.52	3.08	14	.008
SaO2 3rd day	95.40	15	2.13	0.55			
SaO2 2nd day	94.50	10	2.55	0.81	−0.85	9	.415
SaO2 3rd day	95.10	10	2.47	0.78

**Table 4:** Results of measurements of arterial blood pressure (systolic (s) and diastolic (d) ), pulse, and saturation in PVB group on particular days

Statistics for dependent groups					t- test	Degrees of freedom	p
Comparison of hemodynamic parameters between particular days after surgery in PVB group	Mean	N	Standard deviation	Standard error of the mean			
a blood pressure-s- (every 4 h) 1st day	122.10	30	11.25	2.05	−0.10	29	.924
a blood pressure-s- (every 4 h) 2nd day	122.37	30	13.31	2.43			
a blood pressure-s- (every 4 h) 1st day	121.92	24	11.06	2.26	−2.13	23	.044
a blood pressure-s- (every 4 h) 3rd day	129.17	24	15.96	3.26			
a blood pressure-s- (every 4 h) 2nd day	121.33	24	13.80	2.82	−2.17	23	.041
a blood pressure-s- (every 4 h) 3rd day	129.17	24	15.96	3.26			
b blood pressure-d- (every 4 h) 1st day	69.30	30	8.89	1.62	−0.46	29	.650
b blood pressure-d- (every 4 h) 2nd day	70.07	30	10.79	1.97			
b blood pressure-d- (every 4 h) 1st day	68.38	24	8.83	1.80	−2.05	23	.050
b blood pressure-d- (every 4 h) 3rd day	72.50	24	10.16	2.07			
b blood pressure-d- (every 4 h) 2nd day	68.88	24	11.13	2.27	−1.87	23	.075
b blood pressure-d- (every 4 h) 3rd day	72.50	24	10.16	2.07			
pulse 1st day	81.80	30	10.53	1.92	−1.54	29	.136
pulse 2nd day	84.07	30	8.63	1.58			
pulse 1st day	81.17	24	10.38	2.12	−1.37	23	.183
pulse 3rd day	84.04	24	9.93	2.03			
pulse 2nd day	84.21	24	8.09	1.65	0.13	23	.896
pulse 3rd day	84.04	24	9.93	2.03			
SaO2 1st day	97.28	17	1.83	0.44	3.86	16	.001
SaO2 2nd day	95.18	17	2.30	0.56			
SaO2 1st day	97.94	7	2.04	0.77	4.44	6	.004
SaO2 3rd day	95.71	7	0.95	0.36			
SaO2 2nd day	94.75	4	3.20	1.60	−0.42	3	.703
SaO2 3rd day	95.25	4	0.96	0.48

**Table 5:** Results of measurements of arterial blood pressure (systolic (s) and diastolic (d) ), pulse, and saturation in TEA group on particular days

Statistics for dependent groups					t-test	Degrees of freedom	p
Comparison of hemodynamic parameters between particular days after surgery in TEA group	Mean	N	Standard deviation	Standard error of the mean			
a blood pressure-s- (every 4 h) 1st day	119.13	30	13.50	2.47	−0.34	29	.734
a blood pressure-s- (every 4 h) 2nd day	120.00	30	16.43	3.00			
a blood pressure-s- (every 4 h) 1st day	120.35	23	14.63	3.05	−1.10	22	.284
a blood pressure-s- (every 4 h) 3rd day	123.91	23	12.92	2.69			
a blood pressure-s- (every 4 h) 2nd day	120.61	23	17.71	3.69	−1.03	22	.314
a blood pressure-s- (every 4 h) 3rd day	123.91	23	12.92	2.69			
b blood pressure-d- (every 4 h) 1st day	66.83	30	7.64	1.40	−0.50	29	.622
b blood pressure-d- (every 4 h) 2nd day	67.47	30	7.64	1.40			
b blood pressure-d- (every 4 h) 1st day	67.22	23	7.94	1.65	−2.37	22	.027
b blood pressure-d- (every 4 h) 3rd day	72.09	23	8.81	1.84			
b blood pressure-d- (every 4 h) 2nd day	67.09	23	8.46	1.76	−2.39	22	.026
b blood pressure-d- (every 4 h) 3rd day	72.09	23	8.81	1.84			
pulse 1st day	79.93	30	10.76	1.96	−3.33	29	.002
pulse 2nd day	84.56	30	10.07	1.84			
pulse 1st day	79.96	23	10.34	2.16	−2.57	22	.018
pulse 3rd day	85.09	23	9.83	2.05			
pulse 2nd day	83.43	23	10.32	2.15	−1.07	22	.296
pulse 3rd day	85.09	23	9.83	2.05			
SaO2 1st day	97.12	20	1.61	0.36	3.71	19	.002
SaO2 2nd day	95.20	20	2.50	0.56			
SaO2 1st day	96.93	8	2.03	0.72	1.53	7	.169
SaO2 3rd day	95.13	8	2.85	1.01			
SaO2 2nd day	94.33	6	2.34	0.95	−0.70	5	.516
SaO2 3rd day	95.00	6	3.22	1.32			

Table 6 presents comparison of other tested parameters in PVB and TEA groups.

**Table 6:** Other tested parameters in PVB and TEA groups

Tested parameter	Group PVB	Group TEA	p	Minimum	Maximum
Number of days of hospitalization	7.47 (SD 3.7)	6.3 (SD 1.95)	0.15	3	23
Time of surgical procedure expressed in minutes	91.67 (SD 28.2)	98.67 (SD 37.87)	0.646	45	185
Time of maintaining pleural drain after the surgical procedure expressed in days	5.13 (SD 4.17)	4.93 (SD 1.84)	0.736	2	23

### Additional pain relievers

There were additional pain relievers (morphine, metamizole, ketoprofen, paracetamol) with basic analgesia method (PVB or TEA), while in the case of some patients combination of these drugs was used (Table 7).

**Table 7:** Use of additional pain relievers in particular gropus

Drug (24-h doses)	PVB group	TEA group	p
Morphine (30 mg)	6 persons (20%)	3 persons (10%)	0.28
Other pain relievers Paracetamol (4 g) Ketoprofen (200 mg)	16 persons (53.3%)	9 persons (30%)	0.07
Metamizole (3 g)	15 persons (50%)	22 persons (73.3%)	0.06

Morphine and other pain relievers, such as paracetamol or ketoprofen were used more often in PVB group, while metamizole was more often used in TEA group; however, there was no statistical difference in terms of the use of additional pain relievers between the groups.

### Complications

Complications were observed in 11 persons (18.3%). In 4 persons (13.3%) from the PVB group, and in 7 persons (23.3%) from the TEA group. The difference between the groups was not statistically significant (*p* = 0.32). Leaking of regional analgesia medications outside the epidural catheter, catheter coming out from the paravertebral area, fever, retention of secretion in the respiratory tract, respiratory failure have been observed in PVB group. In the TEA group complications included, among others, retention of secretion in the respiratory tract, paroxysmal atrial fibrillation.

Partial spinal anesthesia with symptoms of paraplegia involving all body segments from Th4 downwards, was observed in one patient from TEA group. The patient has not lost consciousness or respiratory efficiency, because upon occurrence of the first symptoms of spinal anesthesia “epidural” infusion of 0.125% bupivacaini was stopped and the epidural catheter was removed. Symptoms of spinal anaesthesia resolved within four hours. MRI of thoracic spine was performed. No damage to the spinal cord was observed. No neurological consequences have been observed in the patient. Upon completion of the treatment, the patient was discharged home in good general condition.

### Pain severity assessment

#### Pain severity assessment using NRS

Groups were compared in terms of pain severity using NRS on three days after the surgery; results are presented in Table 8.

**Table 8:** Comparison of NRS values in particular groups on three subsequent days after the surgery

NRS measurements	PVB mean (SD)	TEA mean (SD)	P
NRS 1st day	4.33 (1.37)	3.93 (1.18)	*P* = 0.206
NRS 2nd day	3.05 (0.88)	3.11 (1.27)	*P* = 0.935
NRS 3rd day	2.55 (1.06)	2.37 (1.25)	*P* = 0.496

No statistically significant differences in NRS values between PVB and TEA groups have been observed on three subsequent days after the surgery.

NRS values measured every 4 hours have been compared for both groups altogether and for each of the groups separately.

On the first day NRS value for both groups altogether was 4.13 with standard deviation of 1.28; on the second day 3.08 with standard deviation of 1.08, and on the third day 2.46 with standard deviation of 1.15. For both groups altogether, NRS values on the first day compared to the values obtained on the second day and values obtained on the first day compared to the values obtained on the third day, as well as values obtained on the second day compared to the values obtained on the third day were statistically significantly lower (*p* = 0.00).

While comparing NRS values in the PVB group, on the first and the second day and on the first and the third day, as well as on the second and on the third day, statistically significant decreasing of NRS values has been observed, *p* = 0.000, *p* = 0.000 and *p* = 0.008, respectively.

While comparing NRS values in the TEA group, on the first and the second day and on the first and the third day, as well as on the second and on the third day, statistically significant decreasing of NRS values has been observed, *p* = 0.001, *p* = 0.000 and *p* = 0.001, respectively.

No statistically significant differences in assessment of particular statements in both groups have been observed, except for the assessment of the lowest level pain within the last 24 h (*p* = 0,019).

Table 9 presents results obtained in particular groups in Clinical Quality Indicators in Postoperative Pain Management scale and comparison of both groups.

**Table 9:** Results obtained in particular groups in Clinical Quality Indicators in Postoperative Pain Management scale and comparison of both groups

	Group														Mann - Whitney U test	p
	Paravertebral block				Epidural analgesia				Total							
	Mean	Median	N	Standard deviation	Mean	Median	N	Standard deviation	Mean	Median	N	Standard deviation	Minimum	Maximum		
Before my operation I was told about the type of pain treatment I would be offered after surgery	4.90	5.00	30	0.31	4.93	5.00	30	0.25	4.92	5.00	60	0.28	4.00	5.00	435.00	.643
After my operation I talked with a nurse about how I wanted my pain to be treated	4.50	5.00	30	1.17	4.53	5.00	30	1.17	4.52	5.00	60	1.16	1.00	5.00	437.00	.776
I received support or help in finding a comfortable position in bed to help avoid pain	4.63	5.00	30	0.85	4.57	5.00	30	1.04	4.60	5.00	60	0.94	1.00	5.00	441.50	.852
I was given the opportunity for peace and quiet so I could sleep at night	4.43	5.00	30	0.97	4.53	5.00	30	0.82	4.48	5.00	60	0.89	2.00	5.00	432.00	.737
Even if I did not always ask for it, I was given pain medication	4.73	5.00	30	0.64	4.87	5.00	30	0.43	4.80	5.00	60	0.55	3.00	5.00	418.00	.423
The staff asked me about the pain I had when I breathed deeply, sat up, or moved around	4.83	5.00	30	0.53	4.93	5.00	30	0.25	4.88	5.00	60	0.42	3.00	5.00	433.00	.600
To determine my level of pain a member of the staff asked me to pick a number between 1 and 10 (or make a mark on a straight line) at least once every morning, after- noon, and evening	4,80	5,00	30	0,81	4,93	5,00	30	0,25	4,87	5,00	60	0,60	1,00	5,00	448,00	.945
The nurses helped me with pain treatment until I was satisfied with the effects of pain relief	4,87	5,00	30	0,51	4,87	5,00	30	0,35	4,87	5,00	60	0,43	3,00	5,00	424,00	.460
I had a pleasant room	4,67	5,00	30	0,76	4,93	5,00	30	0,25	4,80	5,00	60	0,58	2,00	5,00	387,00	.115
There have been enough nurses on duty for some- one to respond quickly to my request for pain relief	4,77	5,00	30	0,63	4,70	5,00	30	0,60	4,73	5,00	60	0,61	3,00	5,00	411,50	,398
When nurses come on duty, they know ‘everything’ about how much pain I have had and the pain treatment I have received	4,87	5,00	30	0,51	4,87	5,00	30	0,43	4,87	5,00	60	0,47	3,00	5,00	437,00	,688
The nurses are knowledgeable about how to relieve my pain	4,93	5,00	30	0,37	4,93	5,00	30	0,25	4,93	5,00	60	0,31	3,00	5,00	436,00	,584
The nurses believe me when I tell them about my pain	4,90	5,00	30	0,40	4,93	5,00	30	0,37	4,92	5,00	60	0,38	3,00	5,00	435,50	,570
The nurses and doctors have cooperated in treating my pain	4,93	5,00	30	0,37	4,93	5,00	30	0,25	4,93	5,00	60	0,31	3,00	5,00	436,00	,584
Highest level of pain within the last 24 h	6,03	6,50	30	2,24	5,30	5,00	30	2,59	5,67	5,00	60	2,43	1,00	10,00	368,00	,221
Lowest level of pain within the last 24 h	1,57	2,00	30	1,19	0,93	0,00	30	1,48	1,25	1,00	60	1,37	0,00	6,00	300,00	,019
Current pain	2,20	2,00	30	1,52	1,80	1,50	30	2,07	2,00	2,00	60	1,81	0,00	9,00	353,50	,144
Satisfaction with pain management	9,03	10,00	30	1,67	9,07	10,00	30	1,60	9,05	10,00	60	1,62	2,00	10,00	436,50	,819

## Discussion

Methods of regional analgesia are often used in treatment of pain after the chest area surgeries. Currently, TEA is the method of choice. Even though it is considered the method of choice it has many contraindications and side effects. Alternative method of local analgesia in thoracic surgeries is PVB above all [15]. Many authors consider PVB a method with similar effectiveness in pain management to TEA, but with more beneficial side effects profile [10, 16, 17].

### Hemodynamic parameters - arterial blood pressure and heart rate

Systolic and diastolic pressure on particular three days after the surgery were similar in both groups. Statistically significant increase of systolic and diastolic pressure values between the first and the third day, and the second and the third day, as well as increase in the heart rate values between the first and the second day and the first and the third day has been observed for both groups analysed altogether. Statically significant increase of systolic pressure values between the first and the third day and the second and the third day as well as increase of the diastolic pressure values between the first and the third day have been observed in the PVB group. Statistically significant increase of diastolic pressure values between the first and the third day and the second and the third day after the surgery was observed in the TEA group. There was no statistically significant difference in the mean values of heart activity between particular days after the surgery in PVB group, while in TEA group statistically significant increase of mean values of heart activity has been observed between the first and the second day and the first and the third day after the surgery; however, on particular days after the surgery both groups were not statistically significantly different in respect of heart rate. Egyptian authors observed significant decrease in arterial blood pressure values in TEA group compared to patients treated with PVB. Similarly, Davies et al. and authors from India stated that TEA causes more evident arterial blood pressure decrease compared to PVB, what results from the fact that TEA has a greater impact on sympathetic system compared to PVB [10, 16, 18]. Studies performed by Szebla et al. also showed non-substantial impact of administration of medicines used for conduction anesthesia to the paravertebral area in the thoracic segment on the basic hemodynamic parameters [19].

### Respiratory system functions - arterial blood saturation

Statistically significant decrease in arterial blood saturation between the first and the second day after the surgery and between the first and the third day was observed in the PVB group. Statistically significant decrease in saturation in TEA group was observed between the first and the second day after the surgery. On respective days the groups showed no statistically significant difference in respect of saturation values changes. For both groups altogether, statistically significant decrease in saturation values were observed between the first and the second day and the first and the third day after the surgery. These observations differ from the ones of Richardson et al. (Richardson et al., 1999), where saturation in PVB group was significantly higher in the follow-up period exceeding 48 hours than in the case of TEA group [9].

### Additional pain relievers

Additional pain relievers were used in both study groups. Morphine, metamizole and other pain relievers, mainly acetaminofen or ketoprofen, were used in the same administration.

In the case of PVB group morphine was twice as likely often and medicines from other groups were slightly less often used compared to TEA group, while metamizole was more often used in TEA group compared to PVB group. No statistically significant differences have been observed between the groups in terms of the need to use additional pain relievers; however, patients from PVB group needed them a bit more often. Authors from India have similar observations - their study showed no statistically significant difference in terms of use of Morphine (it was the only one additional drug) between PVB and TEA groups [18]. In the study conducted by Messina et al., morphine was statistically significantly more often used in PVB group than in TEA group, while in the study performed by Pintarica et al., the need to use morphine was similar in PVB and TEA groups [20, 21]. Similarly, the study conducted by Okajima et al. showed no statistically significant difference in terms of use of additional pain relievers between PVB and TEA group (*p* = 0.26) [11]. In the study conducted by Komatsu, 17.6% of patients who were treated with PVB needed additional pain relievers [22].

### Complications

In our own studies complications were more often observed in TEA group than in PVB group. In PVB group complications were observed in 4 persons, i.e. in 13.3% of the group, while in TEA group complications were observed in 7 persons, i.e. in 23.3%; however the difference in respect of frequency of complications between the study groups was not statistically significant. Complications in PVB group were more often related to catheter and included, among others, leaking of drug outside the catheter or catheter coming out, rather than general complications (e.g. decrease in arterial blood pressure).

In TEA group there were no complications such as hematoma or epidural empyema or damage to nerves. Partial spinal anesthesia with symptoms of paraplegia involving all body segments from Th4 downwards was observed in one patient from TEA group. Symptoms of spinal anesthesia resolved within four hours. Many authors, among others Davies et al. or Baidya et al., emphasize that PVB has in general less complications than TEA, what involves less complications such as decrease in arterial blood pressure, hypoxia, nausea and vomiting or postoperative respiratory failure or pneumonia [1, 10]. Japanese authors in their studies involving patients treated with PVB observed, among others, such complications as atrial fibrillation or respiratory failure requiring mechanical ventilation, but these were single cases [23]. Similarly – also in single cases - occurrence of atrial fibrillation or respiratory failure among the treated patients was observed.

### Pain severity assessment

In the conducted studies we have observed comparable severity of pain in both groups. In the studies conducted by Szebla and Machała, who also used NRS to compare pain in PVB and TEA groups, NRS values in both groups were similar and the result was below 3 [8]. Tsuboshima et al. analyzed pain ailments in patients undergoing thoracoscopic surgeries of idiopathic pneumothorax, who were treated with PVB. Pain ailments were assessed using NRS - average NRS value after 6 hours amounted to 2.3 point, after 24 hours after the surgery it was 2.2 points, and after 48 hours it was 1 point - it was less than in our studies, what could result from the use of minimally invasive method and from the smaller extent of the surgery [24].

Majority of authors assessing postoperative pain uses VAS. In the study conducted by Richardson et al., for example, VAS value was statistically significantly lower in PVB group than in TEA group (*p* = 0.02). While, in the meta-analysis of Ding et al. there were no statistically significant differences in VAS values between PVB and TEA groups at 4-8 hours after the surgery (*p* = 0.19), 24 hours after the surgery (0.77) and 48 hours after the surgery (*p* = 0.19) [3, 9].

In the study conducted by Japanese authors, there were no differences between PVB and TEA groups in terms of pain ailments assessed using VRS (Verbal Rating Scale) [11].

### Number of days of hospitalization

In the conducted studies it was assessed if the type of regional postoperative analgesia in chest surgeries affects the number of days of the patient’s stay at the hospital. Statistically significant difference in the number of days of hospitalization between PVB and TEA groups has not been observed.

However, Elsayed et al. observed statistically significant difference between the time of hospitalization of patients who were treated with PVB compared to TEA patients (*p* = 0.008) of 1 day (6 days vs. 7 days) - time of hospitalization of patients treated with PVB was 1 day shorter and based on this observation they suggested that PVB can become an element of the so called fast track management in chest surgeries. However, this requires further research and improvement of PVB technique [25].

### Assessment of patients’ satisfaction with pain management

Assessment of patients’ satisfaction with pain management was performed based on the Clinical Quality Indicators in Postoperative Pain Management scale, translated into Polish and adopted to local conditions [13, 14]. Among aspects indicated on the said scale, methods of analgesia being compared in the study, i.e. PVB and TEA, were statistically significantly different only in respect of the category “the lowest level of pain within the last 24 h” in the scale of 0-10 points. Result in TEA group was statistically significantly lower than the one in PVB group (*p* = 0.019). In the case of other criteria, PVB and TEA groups were not statistically significantly different. In the study conducted by Okajima et al., no statistically significant difference in respect of the general patient’s satisfaction with treatment (authors used another scale than the one used in our study) was observed between PVB and TEA group (*p* = 0.26) [11].

## Conclusions

1. PVB and TEA are not significantly different in terms of postoperative pain management and the need to use additional pain relievers.
2. In the assessment of postoperative pain management quality, both analyzed methods are statistically significantly different in favor of TEA only in the category of “the lowest level of pain within the last 24 hours” of the Quality of Indicators in Postoperative Pain Management scale.
3. No significant differences have been observed in respect of occurrence of complications between the two compared methods.
4. TEA seems to be better in managing pain after thoracotomy on the basis of our results, however both methods are comparable.

## Abbreviations

MRI: Magnetic resonance imaging
NRS: Numeric rating scale
PVB: Paravertebral block
TEA: Thoracic epidural analgesia
VAS: Visual analog scale
VATS: Video-assisted thoracoscopic surgery
